# Alternative preparation of propolis extracts: comparison of their composition and biological activities

**DOI:** 10.1186/s12906-015-0677-5

**Published:** 2015-05-27

**Authors:** Loreta Kubiliene, Virginija Laugaliene, Alvydas Pavilonis, Audrius Maruska, Daiva Majiene, Karolina Barcauskaite, Raimondas Kubilius, Giedre Kasparaviciene, Arunas Savickas

**Affiliations:** Department of Drug Technology and Social Pharmacy, Lithuanian University of Health Sciences, A.Mickeviciaus 9, LT-44307 Kaunas, Lithuania; Department of Biology, Vytautas Magnus University, Vileikos 8, LT-44404 Kaunas, Lithuania; Department of Microbiology, Lithuanian University of Health Sciences, Eiveniu 4, LT-40009 Kaunas, Lithuania; Institute of Cardiology, Lithuanian University of Health Sciences, Sukileliu 17, LT-5016 Kaunas, Lithuania

**Keywords:** Water, Oil, Ethanol, PEG, Extract of propolis, Polyphenols, Radical scavenging activity, Antimicrobial activity

## Abstract

**Background:**

Propolis is the bee product noted for multiple biological effects, and therefore it is widely used for the prevention and treatment of a variety of diseases. The active substances of propolis are easily soluble in ethanol. However ethanolic extracts cannot be used in treatment of certain diseases encountered in ophthalmology, pediatrics, etc. Unfortunately, the main biologically active substances of propolis are scarcely soluble in water, oil and other solvents usually used in pharmaceutical industry. The aim of this study was to investigate chemical composition, radical scavenging and antimicrobial activity of propolis extracts differently made in nonethanolic solvents.

**Methods:**

Total content of phenolic compounds in extracts was determined using Folin-Ciocalteu method. Chemical composition and radical scavenging activity of extracts were determined using HPLC system with free radical reaction detector. Antimicrobial activity of examined preparations was evaluated using the agar-well diffusion assay.

**Results:**

Total amount of phenolic compounds in extracts made in polyethylene glycol 400 (PEG) and water mixture or in PEG, olive oil and water mixture at 70 °C was comparable to that of ethanolic extract. Predominantly identified compounds were phenolic acids, which contribute ca. 40 % of total radical scavenging activity.

Investigated nonethanolic extracts inhibited the growth and reproduction of all tested microrganisms. Antimicrobial activity of some extracts was equal or exceeded the antimicrobial effect of ethanolic extract. Extracts made in pure water or oil only at room temperature, contained more than 5 – 10-fold lower amount of phenolic compounds, and demonstrated no antimicrobial activity.

**Conclusions:**

Nonethanolic solvent complex and the effect of higher temperature allows more effective extraction of active compounds from propolis. Concentration of total phenolic compounds in these extracts does not differ significantly from the concentration found in ethanolic extract. Propolis nonethanolic extracts have radical scavenging and antimicrobial activity.

**Electronic supplementary material:**

The online version of this article (doi:10.1186/s12906-015-0677-5) contains supplementary material, which is available to authorized users.

## Background

Propolis and its extracts have long been used for the prevention and treatment of a variety of diseases due to its antibacterial, antiviral, antifungal, antioxidant, anesthetizing, cytostatic, anti-inflammatory, and immune-strengthening, hepatoprotective effect, etc. [1, 2]. Among the many biological activities of propolis extracts, antimicrobial effects have been widely reported. Ethanolic extracts of propolis have been found to be effective against a broad range of bacteria, especially against gram-positive bacteria species [3]. The antimicrobial activity of European propolis has been related with contained phenolics, flavonoids and derivatives of caffeic acid [4-6].

The most popular technique for the production of propolis extracts is ethanol extraction. This method is suitable for obtaining low-wax propolis extracts rich in biologically active compounds [7]. Although extraction with ethanol is a simple and effective method, it has disadvantages such as strong residual flavor, limitations of application in cosmetics and pharmaceutical industry: for example, in medicine ethanol extracts are not suitable for treatment of some diseases in ophthalmology, otorhinolaryngology, pediatrics, or in cases of alcohol intolerance. According to various authors, development of propolis preparations based on effective nonethanolic extraction techniques is desirable.

There is little data on the production of water solutions of propolis. Biologically active substances mostly have low solubility in water, and the amount of phenolic compounds in water extracts is 10-fold lower than in ethanolic extracts [8, 9]. Therefore, it is important to find effective co-solvents which increase the solubility of these substances in water.

There are only a few articles dealing with preparation of propolis oily extracts, even if they are/could be the optimal form of propolis for the introduction into dermatological pharmaceuticals and cosmetic preparations [10]. The solubility of biologically active substances in propolis oily extracts is as poor as in water extracts, therefore further studies on water and oil extraction of propolis are also needed.

According to literature data, nonethanolic propolis extracts and their major compounds possess higher pharmacological activity, as compared to ethanolic extracts [11]. Propolis extract in water suppresses the generation of free radicals more effectively, as compared to ethanolic extract of propolis [12]. Moreover, water soluble derivatives of propolis and its polyphenolic compounds significantly reduce the growth and proliferation of tumour cells [13]. It was also reported that phenolic compounds present in oil extract of Brazilian propolis have the effective antimicrobial and antitumoral activity [14]. Nevertheless, water and oily extracts have not been sufficiently studied yet, therefore it is important to investigate the chemical composition and biological activity of nonethanolic propolis extracts.

The aim of this study was to investigate the chemical composition and radical scavenging activity of differently prepared propolis extracts, and to determine antimicrobial activity of these solutions.

## Methods

### Propolis raw material and preparation of propolis extracts

Propolis was collected in Lithuania during September of 2011. The apiary was (is) near deciduous (hardwoods) forest. Prior to analysis, propolis samples were kept at room temperature in the dark. Crude propolis was grounded into powder and macerated in different solvents by shaking. The list of propolis solvents, temperature modes and time of extraction is provided in Table 1. After extraction, extracts of propolis were filtered through Whatman No. 1 filter paper. Solutions are clear, yellow, viscous (with PEG and oil) liquids and remain stable when stored, i.e. the colour remains unchanged, no precipitate is observed and they do not turn white.

**Table 1:** Conditions of preparation of different propolis extracts

Sample	Composition	Extraction temperature	Extraction time	Total amount of phenolic compounds mg/mL GAE
W_1_	Propolis 10 g; Water ad 100 mL	Room temperature	5 h	1.6 ± 0.4 *
W_2_	Propolis 10 g; PEG 400 20 g; Water ad 100 mL	70° C	15 min.	10.7 ± 1.2
A_1_	Propolis 10 g; Olive oil ad 100 mL;	Room temperature	5 h	0.5 ± 0.2 *
A_2_	Propolis 10 g; PEG 400 20 g; Olive oil 50 g; Water ad 100 mL	70° C	15 min.	9.5 ± 1.3
EEP	Propolis 10 g; ethanol 70 % ad 100 mL	Room temperature	5 h	12.7 ± 1.2

Number or experiments - 3–5

* - P < 0.05 *vs* EEP

### Spectrophotometric evaluations

Total content of phenolic compounds in extracts was determined spectrophotometrically using the Folin-Ciocalteu method [15]. 0.1 mL extract was mixed with 2.5 mL distilled water, 0.1 mL of Folin-Ciocalteu reagent and 0.5 mL of 20 % sodium carbonate. The colour was developed for 2 h at room temperature and the absorbance was measured at 760 nm wavelength. Total phenolics content was estimated using calibration curve of gallic acid, concentration range 0,016 – 1,040 mg/mL. The total phenolic content was expressed in mg of gallic acid equivalents (GAE) per mL of extract.

For extraction of phenolic compounds from oily samples liquid-liquid extraction was carried out. Tested oily solution and the same amount of MeOH were added in a separation funnel. The funnel was shaken for 15 min for the extraction of phenolic compounds. The metanolic fraction was then withdrawn and stored in a test tube.

Total radical scavenging activity of propolis extracts was evaluated using method based on bleaching of solution of synthetic free radical DPPH [16]. Bleaching of DPPH free radical solution is one of the most popular techniques for evaluation of radical scavenging activity. 0.077 mL extract of propolis was mixed with 3 mL DPPH solution. DPPH solution was prepared by the following procedure: 10 mg of DPPH reagent was dissolved in acetonitrile and methanol mixture (1:1 v/v) and added 250 mL 100 mM acetate buffer with pH 5.5 in order to obtain DPPH solution concentration 20 mg/L. Sample and DPPH radical solution was shaken well and incubating for 15 min in ambient temperature in dark. Absorbance was measured at 515 nm wavelength. Obtained results were calculated according to the formula:

$$ \left[\frac{A_{Bl} - {A}_{Sp}}{A_{Bl}}\right]\cdotp 100\% $$

*A*_*Bl*_absorption of blank solution

*A*_*Sp*_absorption of sample solution.

To measure the absorbance, a spectrophotometer Milton Roy Spectronic 1201 (Milton Roy Company, USA) was used.

### Instrumentation and conditions of chromatographic analysis

The chemical composition and radical scavenging activity of propolis extracts were determined using high performance liquid chromatography system with free radical reaction detector, comprised of gradient pump (VARIAN 9012, Solvent Delivery System, USA), gradient mixer (SP8500, Spectra-Physics, USA), precolumn (LiChrospher RP-18e, 4 × 4 mm) and LiChrospher RP-18, d_p_ = 5 *μm*, 125 × 4 mm column. Separated compounds were detected by UV detector Linear 206 PHD (Linear Instruments, USA). DPPH solution was introduced to the system by radical reagent pump (BISCHOFF HPLC pump, Germany). The signal of radical scavenging action was detected by visible light detector Linear UVIS 200. Chromatographic parameters were registered and calculated via the Clarity Lite software (DataApex, Czech Republic).

For chromatographic separation of propolis compounds two mobile phase components were used: A (water with 0.05 % TFA additive) and B (CH_3_OH with 0.05 % TFA additive). DPPH reagent was prepared at a concentration of 20 mg/L in complex solvent consisted of acetonitrile: methanol: 0.1 M citrate buffer, pH 7.6 (volume ratio 25:25:50). Gradient elution was carried out. Gradient was as follows: 0 – 25 min. from 20 to 95 % and then back to 20 % of B. The flow rate of mobile phase and DPPH solution was 0.75 mL min^−1^. The injected sample volume was 20 *μ* L. Detection of UV signal was made at 254 nm, while detection of radical scavenging action was observed at 517 nm. Peaks of analytes in the chromatograms were identified using standard solutions. Following standard compounds were used for identification of polyphenols in the extracts of propolis: trans-p-coumaric acid (99.9 %, Chromadex, USA), naringenin (99.9 %, Chromadex, USA), chlorogenic acid (>99.9 %, Sigma -Aldrich, USA), caffeic acid (>98,9 %, Sigma-Aldrich, Switzerland), ferullic acid (99.9 %, Chromadex, USA), rutin (≥95 %, Sigma-Aldrich, USA), galangin (≥95 %, Sigma-Aldrich, USA).

Amount of chromatographically determined compounds were calculated using rutin standard calibration curve. Rutine calibration curve should have linear dependence. RSA was calculated using linear equation: y = 8394.4x. Obtained results were expressed in mg/mL rutine equivalent (RE). Correlation coefficient R^2^ = 0.9869. In our research work rutine calibration curve from 0.05 mg/mL to 0.25 mg/mL was used. Extracts were diluted with solvent and obtained results were recalculated assessing the dilution factor. RSA of individual compound was calculated using proportion according total peak area. Total DPPH chromatogram peak area was equal to 100 % and identified compounds area respectively was calculated using proportion.

### Antimicrobial activity

Antimicrobial activity of propolis was established for 6 test strains of microorganisms: Gram-positive bacteria (*Staphylococcus aureus* ATCC 25923), Gram-positive spore-forming bacteria (*Bacillus cereus* ATCC 8035), Gram-negative bacteria (*Escherichia coli* ATCC 25922, *Pseudomonas aeruginosa* ATCC 27853, *Klebsiella pneumoniae* ATCC 33499), and yeast (*Candida albicans* ATCC 60193).

The evaluation of the antimicrobial activity of the investigated solutions was performed using the agar-well diffusion assay according to CLSI, 2008 [17]. Bacterial cultures were grown for 18 h at the temperature of 37 °C on Slant Tryptone Soya Agar (BBL, Cockeysville, USA). The grown cultures were washed off the agar using sterile saline solution and the cell suspensions were adjusted according to McFarland No. 0.5 standard. The yeast fungus culture grew for 48 h at the temperature of 25 °C on Slant Sabouraud Dextrose Agar (BBL, Becton Dickinson and Company, USA). The grown cultures were washed off the agar using sterile saline solution. The cell suspension prepared according to 0.5 McFarland standard.

1 mL of the cell suspension was introduced into dissolved and cooled to 45 °C Mueller-Hinton Agar medium (BBL, Becton Dickinson and Company, USA) and thoroughly stirred. The prepared mixture of the suspension of microbial cells and the medium was poured into 9 cm-diameter glass Petri dishes (30 mL of the suspension per each dish). After the medium hardened, 7 wells (8 mm in diameter) were made in it, and 0.12 mL of examined solutions were poured into the wells.

The antimicrobial effect of investigated solutions on bacterial culture was evaluated after 24 h, and its effect on yeast fungus – after 24–48 h after cultivation. The evaluation was based on the diameter (in mm) of clear zones formed around the wells. If no clear zones were formed around the wells, we concluded that the investigated solution had no antimicrobial effect on the tested culture. Ampicilinum, ceftazidinum, cefuroximum were used as positive control for bacteria strains. Fluconazolum was used as a positive control for fungi. A_2_, W_2_ solvents - as negative control. Number of experiments – 4 – to 6.

### Statistical analysis

Data are presented as means ± standard deviation. Nonparametric methods were applied for making inferences about the data. Differences between mean values in dependent groups were tested using the Wilcoxon matched pairs test. Differences between mean values in independent groups were tested using nonparametric Kruskal-Wallis test with Dunns post-hoc evaluation. The statistical analysis was performed at p < 0.05 using the software package Statistica 1999, 5.5 StatSoft Inc USA.

## Results and Discussion

There is little data on the production, chemical composition and biological activity of water and oily solutions of propolis. There are few biologically active substances of propolis that can be extracted with water, therefore water heating is applied in order to increase the solubility of poorly soluble compounds [2, 18]. For the propolis extraction of poorly soluble in water and oil substances, we used not only extraction at 70 °C temperature, but also additional solvent PEG. PEG is widely used in pharmaceutical industry and it is suitable for a formulation of parenteral drug forms. In parenteral drug forms - maximal concentration of PEG is 30 % [19]. We have chosen a solvent content of 20 % PEG, because these pharmaceutical forms must be sterile (sterile filtration through a 0.22 micrometer pore membrane) - higher PEG concentrations cause significantly more difficult filtration condition.

We investigated the chemical composition and radical scavenging activity of differently prepared nonethanolic propolis extracts, and compared results with respective parameters of ethanolic propolis extract.

Firstly, the total amount of phenolic compounds was determined using Folin- Ciocalteau method. The quantitative analysis showed (Table 1) that total amount of phenolic compounds detected in ethanolic propolis extract (12.7 mg/mL GAE) did not differ significantly from the concentration of phenolic compounds found in A_2_, W_2_ extracts. Extract W_2_, which was made extracting at 70 °C with PEG and water mixture as a solvent, contained the higher amount of phenolic compounds (10.7 mg/mL GAE), whereas a lower amount was detected in extract A_2_, which was made extracting at 70 °C with PEG, olive oil and water mixture as a solvent (9.5 mg/mL GAE). Extracts made in room temperature with water - W_1_ or oil - A_1_ only, contained more than 5–10 fold lower amount of phenolic compounds: 1.6 and 0.5 mg/mL GAE, respectively.

The Folin-Ciocalteau method gives overall comparative results, however determination of different classes of compounds or phenolics is not specific. For the analysis of individual compounds presented in extracts HPLC method was used. HPLC analysis has shown that despite lower overall amount of phenolic compounds, as compared with W_2_, in A_2_ extract flavonoids were also extracted: naringenin, galangin and kaempferol. A comparison of qualitative composition of A_2_ and W_2_ extracts suggests that more different compounds can be extracted using addition of olive oil, which has lower polarity. Addition of PEG and the effect of high temperature statistically significantly increased total amount of phenolic compounds in extracts. HPLC analysis shows that this method enabled successful extraction of phenolic acids: ferulic, trans-p-coumaric, caffeic acids were identified.

Obtained data correspond with results published by other investigators which in water-soluble phase found ferulic, caffeic, p-coumaric acids and quercetin, Artepillin-C® [20, 21]. Our results and other author data suggest that nonethanolic propolis extracts have sufficient amount of phenolic compounds, however further investigations are necessary to improve and validate propolis extraction technology.

In the second series of experiments the radical scavenging activity of differently prepared nonethanolic extracts of propolis was evaluated spectrophotometrically. The lowest activity (20.6 %) possess A_1_, which is made extracting in oil at room temperature and contains the lowest amount of phenolic compounds. Radical scavenging activity of this extract expressed in rutin equivalents was 0.8 mg/mL RE. Higher activity (26.3 %) possess W_1_, which is made extracting at room temperature with pure water as a solvent. Radical scavenging activity of this extract expressed in rutin equivalents was 2.0 mg/mL RE. Addition of PEG and elevation of temperature significantly increased radical scavenging activity of investigated extracts: A_2_ - 58.1 % (3.9 mg/mL RE) and W_2_ - 65.8 % (4.8 mg/mL RE). Data presented in many articles show that radical scavenging activity of EEP correlate with total content of polyphenolic compounds, especially flavonoids [22, 23]. Our results show that investigated ethanolic extract of propolis (EEP) radical scavenging activity expressed in rutin equivalents was 3.5 mg/mL RE.

**Figure 1:**
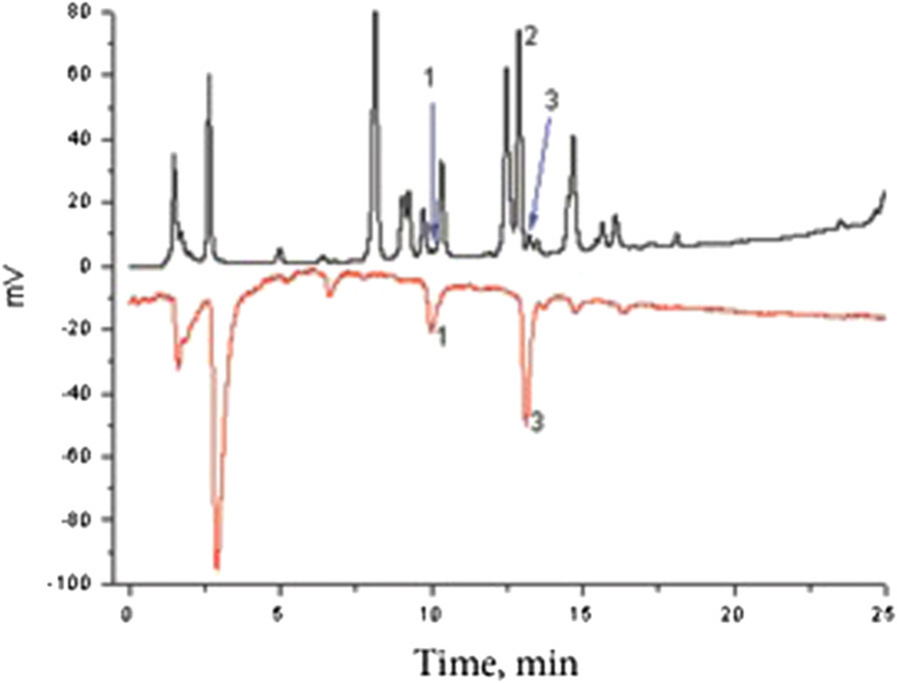
Typical HPLC chromatogram of W_1_ propolis extract. UV chromatogram of water extract with radical scavenging properties reflected in the mirror chromatogram obtained by means of DPPH reaction detection. Identified compounds: 1- caffeic acid; 2 – trans-*p*-coumaric acid; 3 – ferulic acid

The latter results show total radical scavenging activity of investigated extracts. In order to reveal the input of individual compounds present in the extract, the on-line HPLC-DPPH assay was used. This coupled method combines a chromatographic separation of compounds presented in extracts with post-column DPPH scavenging assay and show contribution of individual compounds to antiradical activity of total extract. Figure 1 show typical chromatogram of W_1_ propolis extract with radical scavenging properties reflected in the mirror chromatogram. The main identified compounds are caffeic and ferulic acids. Data, presented in Table 2 demonstrate that the main compounds identified in all extracts were caffeic, trans-p-coumaric, ferulic acids. Flavonoids that are commonly found in the larger part of EEP - determined compounds were identified only in A_2_ extract, and their amounts were small.

**Table 2:** Biologically active compounds identified in different propolis extracts

Compound, RT min.	W_1_	W_2_	A_1_	A_2_	EEP
Caffeic acid, 10.24 min	+	+	+	+	+
Trans-p-coumaric acid, 12.36 min	+	+	+	+	+
Ferulic acid, 12.90 min	+	+	+	+	+
Naringenin, 18.30 min	-	-	-	+	+
Kaempferol, 20.51 min	-	-	-	+	+
Galangin, 23.70 min	-	-	-	+	+

There are only few investigations of chemical composition and antioxidant activity of nonethanolic extracts [24, 25]. According to them water extracts of Brazilian and Turkish 2- propolis have demonstrated scavenging of DPPH, H_2_O_2_, O and OH radicals [2]. Rocha et al. (2013) demonstrated that differently prepared extracts of propolis had significant DPPH radical scavenging activity, but water extract exhibited a higher antioxidant activity than EEP [21]. Our differently prepared extracts were found to contain different amount of polyphenolic compounds and therefore they demonstrated different antiradical activity. A_1_ propolis extract contains the least amount of phenolic compounds and demonstrated relatively low radical scavenging activity. Ferulic acid showed the highest contribution - 51.0 % of total radical scavenging activity of A_1_ extract. W_1_ contains more phenolic compounds and demonstrated higher antioxidant activity. The main identified compounds caffeic and ferulic acids contribute 7.5 % and 15.3 % of total radical scavenging activity, respectively. W_2_ extract which contains the largest amount of polyphenolic compounds showed the highest radical scavenging activity compared to other nonethanolic extracts and EEP. Among its identified compounds, ferulic and caffeic acids contributed 27.6 % and 12.7 % of total radical scavenging activity respectively.

Investigation of propolis extract A_2_ revealed that it contains not only phenolic acids, but also flavonoids. Radical scavenging activity of this extract is less than W_2_ but higher than EEP. Caffeic and ferulic acids possess 7.2 % and 31.6 % of total radical scavenging activity in A_2_ propolis extract, respectively. Although flavonoids were determined in this extract, their amount is small and contribution to total radical scavenging activity is lower than that of phenolic acids.

It is known that phenolic acids demonstrate significant antioxidant effects due to CH = CH-COOH and hydroxyl groups (one - in coumaric and ferulic acids, two – in caffeic acid) [26]. Our obtained results correspond with this assumption and demonstrate that phenolic acids presented in nonethanolic extracts can act as powerful radical scavenging compounds. However, more detailed radical scavenging studies are required to establish the mechanism of this commonly known propolis compounds.

There is a lack of studies that investigate antimicrobial activity of nonethanolic extracts. Moreover in order to estimate the effectiveness of propolis extraction with nonalcoholic solvent mixtures in the last series of studies we investigated the antimicrobial activity of differently prepared nonethanolic extracts using agar-well diffusion method and compared obtained results with that of EEP. Results on the antimicrobial activity (Table 3) showed that propolis extracts with water or oil only (A_1_, W_1_), had no antimicrobial activity, most likely, due to small concentration of active compounds. Extracts W_2_ and A_2_ demonstrated statistically significant inhibition on the growth and multiplication of all tested microrganisms. The effective antimicrobial activity was observed not only against gram-positive bacteria *Staphylococcus aureus* (15.8 mm), spore-forming bacteria Bacillus cereus (17.2 mm) and fungi *Candida albicans* (16.9 mm), but also against gram-negative bacteria –*Escherichia coli*, *Pseudomonas aeruginosa* and *Klebsiella pneumoniae* (no growth of microrganisms in diameter of 16–20.5 mm) as compare with EEP (~15 mm).

**Table 3:** Antimicrobial activity (radius of suppressed microbial growth zone in mm) and DPPH RSA spectrophotometric data (% inhibition) of propolis extracts

Propolis extracts	DPPH RSA (%)	Reference microbial cultures					
		*Staphylococcus aureus* ATCC 25923	*Escherichia coli* ATCC 25922	*Pseudomonas aeruginosa * ATCC 27853	*Klebsiella pneumoniae * ATCC 33499	*Bacillus cereu*s ATCC 8035	*Candida albicans* ATCC 60193
W_1_	26.3	0	0	0	0	0	0
W_2_	65.8	16.2 ± 1.2	16.0 ± 1.4	18.3 ± 1.1	19.4 ± 1.3*	16.9 ± 1.1	19.0 ± 0.5
A_1_	20.6	0	0	0	0	0	0
A_2_	58.1	16.6 ± 1.5	16.2 ± 1.7	17.3 ± 1.4*	20.5 ± 1.1 *	17.8 ± 0.9	20.3 ± 1.2
EEP	52.1	15.8 ± 0.7	14.8 ± 1.0	15.2 ± 1.5	15.4 ± 0.9	17.2 ± 0.6	16.9 ± 1.4
**Positive ** **Control**		Ampicilinum 19 ± 0.5	Ampicilinum 13 ± 1.4	Ceftazidinum 16 ± 1.2	Cefuroximum 18 ± 0.6	Ampicilinum 10 ± 1.0	Fluconazolum 18 ± 1.3

0 - examined preparation has no activity on growth of investigated microbial cultures. Number of experiments – 4–6. * - P < 0.05 *vs* EEP

EEP antimicrobial activity is widely investigated. Literature data demonstrate that antimicrobial activity of this extract is higher against gram-positive bacteria [27, 28]. Several studies demonstrated that propolis antibacterial properties were attributable to its high flavonoid content [29, 30]. Our results regarding ethanolic propolis extract were in concern with our previous and other authors findings and demonstrated higher activity against gram-positive bacteria and fungi (15.8 – 17.2 mm). Gram-negative bacteria were slightly less susceptible to antimicrobial activity EEP (14.8 – 15.4 mm).

Nonethanolic propolis extracts made in mixture of solvents demonstrated statistically significant inhibition on the growth and multiplication of all tested microorganisms. Moreover, antimicrobial activity of these extracts against all investigated microrganisms was equal or exceeded the antimicrobial effect of ethanolic extract. It is important to stress that a higher antimicrobial effect was observed especially in cases with gram-negative bacteria, moreover, A_2_ demonstrated slightly higher antimicrobial effect comparing to W_2_. This effect may result from a wider variety of A_2_ extract chemical composition.

Our results as well as other authors data suggest that nonethanolic propolis extracts have significant antimicrobial effect and that the antimicrobial activities and antioxidant properties were related to the total phenolic contents [10, 28, 31]. However further investigations are necessary to elucidate possibilities and mechanisms of such antimicrobial activity.

In conclusion, present study showed that propolis nonethanolic extracts prepared by using particular solvent complex, had not only significant antioxidant, but also antimicrobial activity, therefore they have high potential in pharmaceutical industry and cosmetics. As ethanolic solutions are not recommended for some diseases and can’t be used in some drug forms (eg. eye, nose medicines etc.), nonethanolic propolis extracts may become a perspective and widely used alternative for extraction of active compounds and development of new preparations. They can also be used as a natural preservative in ecologic food industry.

## Conclusion

Nonethanolic solvent complex on the basis of PEG allows more effective extraction of active compounds from propolis, as compared with extracts containing water or oil only. Concentration of total phenolic compounds in these extracts does not differ significantly from the concentration found in ethanolic extract. Propolis nonethanolic extracts have antioxidant activity resulting mostly from ferulic and caffeic acids. Investigated extracts demonstrate inhibition on the growth and multiplication of all tested microrganisms.

## Abbreviations

PEG: polyethylene glycol 400
DPPH: 1,1-Diphenyl-2-picrylhydrazyl radical
GAE: gallic acid equivalent
RE: rutin equivalent
W_1_: propolis extract in water
W_2_: propolis extract in PEG and water mixture
A_1_: propolis extract in oil
A_2_: propolis extract in oil, PEG and water mixture
EEP: propolis extract in ethanol
TFA: trifluoroacetic acid
RSA: radical-scavenging activity
